# Supplementary material for: The causal relationship between the human gut microbiota and pyogenic arthritis: a Mendelian randomization study
Source: Front Cell Infect Microbiol. 2024 Nov 26;14:1452480. doi: 10.3389/fcimb.2024.1452480 (PMC11629706; doi:10.3389/fcimb.2024.1452480)
Supplement: Supplementary file 1 [file DataSheet1.zip › mendelian test/ebi-a-GCST90027794.csv_forest.pdf]

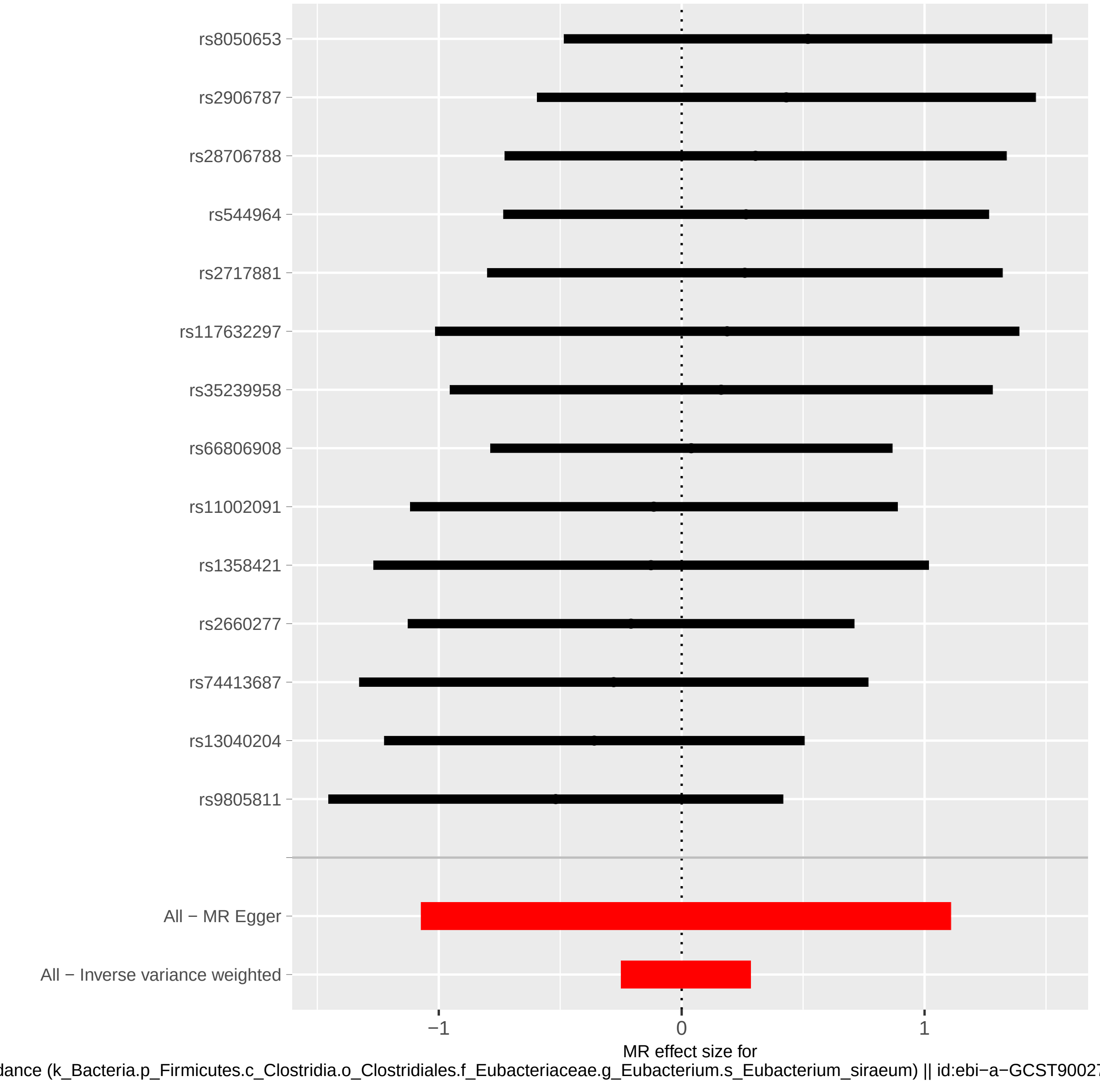

rs8050653
rs2906787
rs28706788
rs544964
rs2717881
rs117632297
rs35239958
rs66806908
rs11002091
rs1358421
rs2660277
rs74413687
rs13040204
rs9805811
All − MR Egger
All − Inverse variance weighted
−1
0
1
MR effect size for
dance (k_Bacteria.p_Firmicutes.c_Clostridia.o_Clostridiales.f_Eubacteriaceae.g_Eubacterium.s_Eubacterium_siraeum) || id:ebi−a−GCST9002
